# Supplementary material for: Radiation-induced extracellular matrix remodelling drives prognosis and predicts radiotherapy response in muscle-invasive bladder cancer
Source: Front Oncol. 2025 Jul 28;15:1616943. doi: 10.3389/fonc.2025.1616943 (PMC12336036; doi:10.3389/fonc.2025.1616943)
Supplement: Supplementary Figure 1 — Fractionated radiotherapy partially mimicking SOC (27.5Gy, 2.75Gy daily over 2 weeks) consistently alters cytokines, glycosaminoglycan binding molecules, growth factors, peptidases and peptidase-regulators expression across all cell lines. Comparative molecular function pathway enrichment analysis (A) of significantly up (fold change >2, p.adj. <0.05) and downregulated (fold change <-2, p.adj. <0.05) ECM proteins for each individual cell line (T24, UMUC3, J82). Cnetplots show specific associations among significantly up and downregulated proteins and their corresponding enrichment terms for T24 (B), UMUC3 (C) and J82 (C). Ratio (A) represents the % of total proteins associated with each term (0–1 scale). A total of n=3 biological repeats were analysed per cell line. [file DataSheet2.docx]

**Supplementary figures**

**
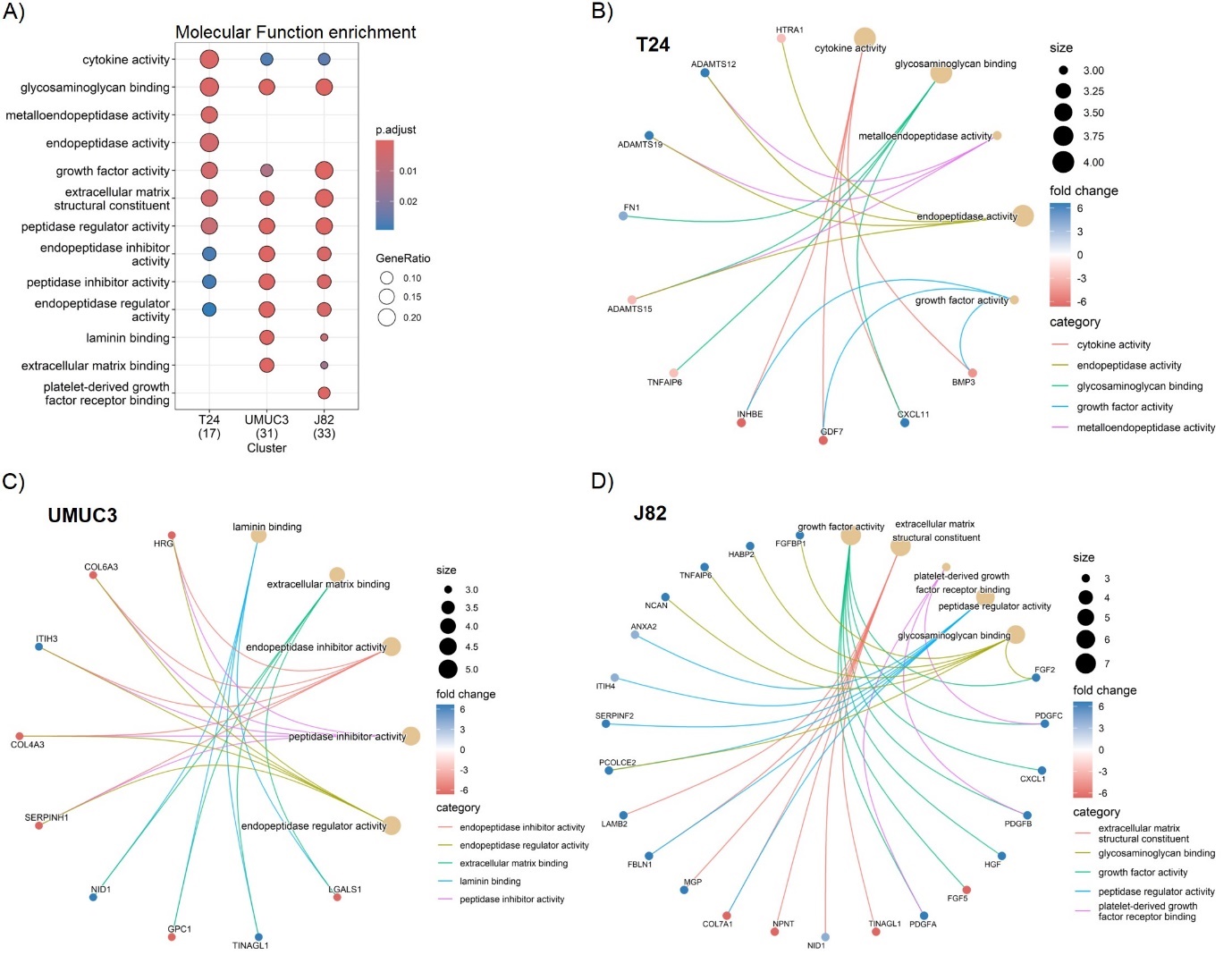
**

**Figure S1: Fractionated radiotherapy partially mimicking SOC (27.5Gy, 2.75Gy daily over 2 weeks) consistently alters cytokines, glycosaminoglycan binding molecules, growth factors, peptidases and peptidase-regulators expression across all cell lines.** Comparative molecular function pathway enrichment analysis (A) of significantly up (fold change >2, p.adj. <0.05) and downregulated (fold change <-2, p.adj. <0.05) ECM proteins for each individual cell line (T24, UMUC3, J82). Cnetplots show specific associations among significantly up and downregulated proteins and their corresponding enrichment terms for T24 (B), UMUC3 (C) and J82 (C). Ratio (A) represents the % of total proteins associated with each term (0 – 1 scale). A total of n=3 biological repeats were analysed per cell line.

**
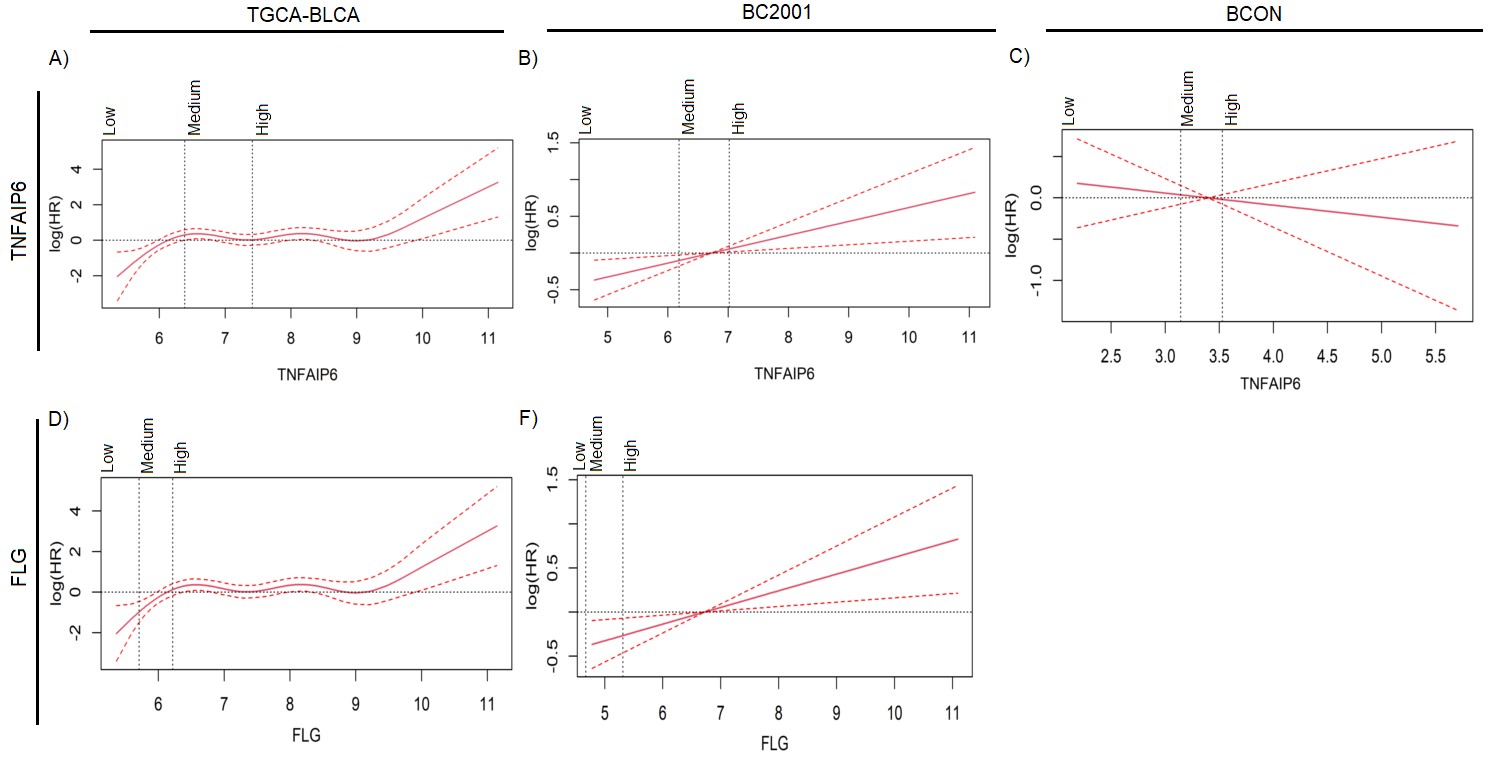
**

**Figure S2: High TNFAIP6 and FLG mRNA expression levels are associated with a linear increase in mortality risk in muscle-invasive bladder cancer (MIBC).** TNFAIP6 (A-C) and FLG (D, M) expression was retrospectively validated in one cystectomy (TCGA-BLCA, n=397) and two radiotherapy (BCON [n=151]; BC2001 [n=313]) cohorts. Cut-off for patients with “High” (>67%), “Medium” (33 – 67%) or “Low” (<33%) hazard risk is based on each cohort’s tertiles median gene expression for TNFAIP6 (J-L) and FLG (M, N) expression. No FLG expression data was available for the BCON cohort.


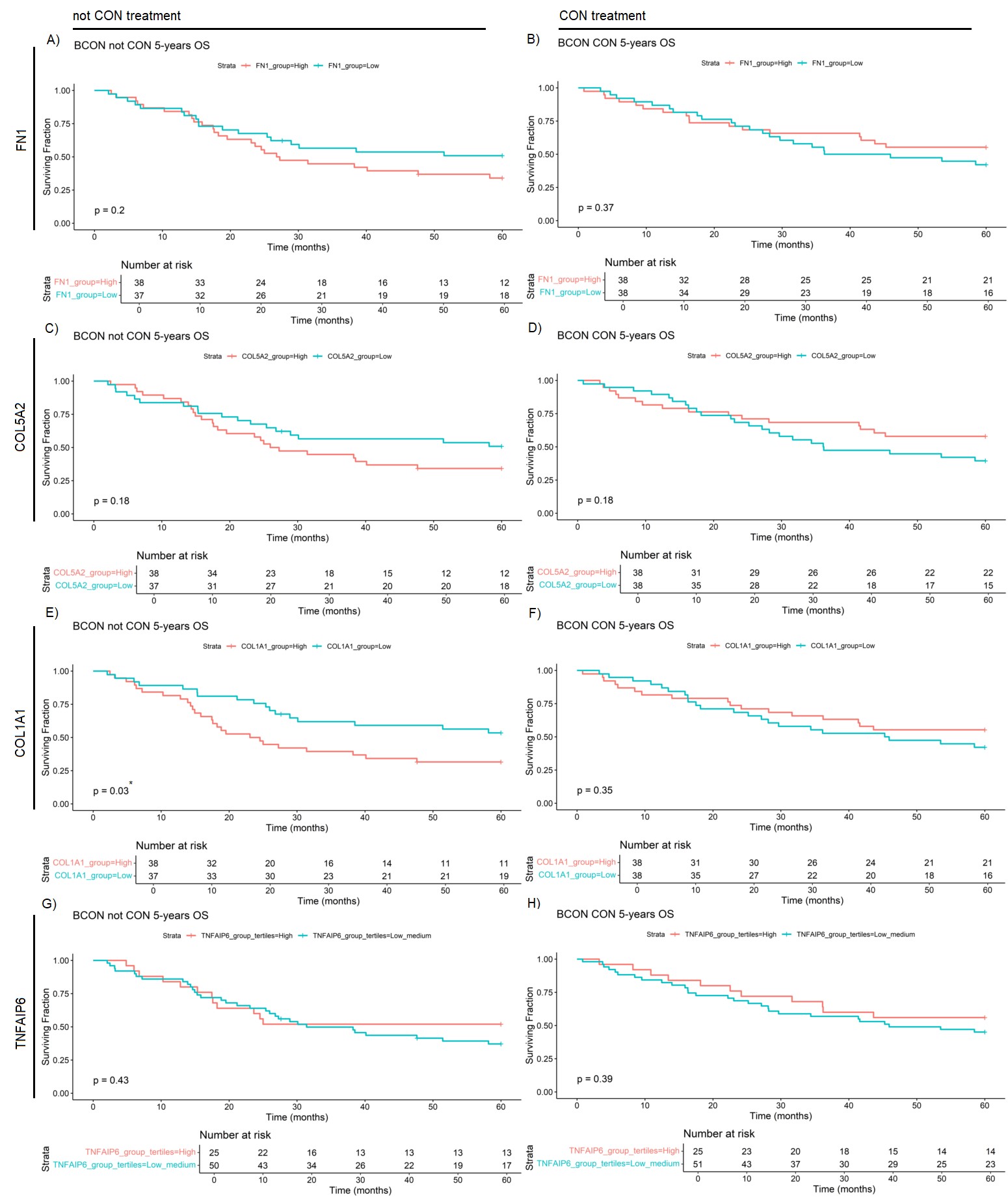


**Figure S3: COL1A1 mRNA expression level is a general prognostic marker for in muscle-invasive bladder cancer (MIBC) patients without carbogen and nicotinamide (CON) hypoxia-modifying treatment.** FN1 (A,B), COL5A2 (C,D), COL1A1 (E,F) and TNFAIP6 (G,H) expression was retrospectively validated in the BCON cohort (n=151) after a split into the two CON treatment arms (n=75 not CON treatment; n=76 CON treatment). COL1A1 expression had significant prognostic value in patients without CON treatment (radiotherapy only) (E). No other gene had significant prognostic value, in either of the CON treatment arms of the BCON cohort. Patients were classified into “High” and “Low” based on each cohort's median gene expression levels for FN1 (A,B), COL5A2 (C,D) and COL1A1 (E,F) analyses. Patients were classified into “High”, “Medium” or “Low” based on each cohort’s tertiles median gene expression for TNFAIP6 (G, H) expression. Significance was defined as p≤0.05.

**
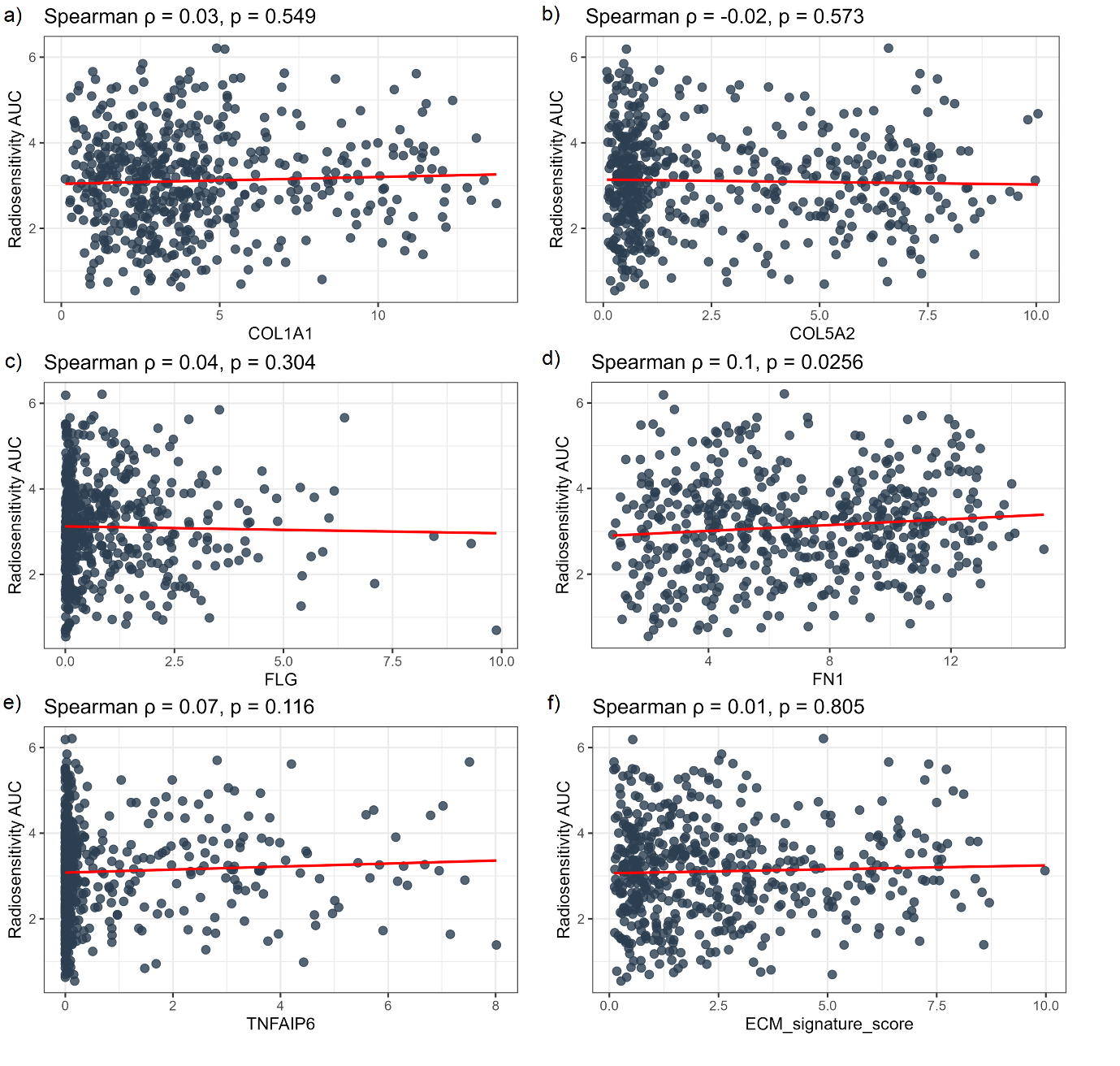
**

**Figure S4. *FN1* expression significantly correlates with radiosensitivity [integral survival (AUC)] across a panel of 535 cancer cell lines.** Pearson correlation was measured between AUC radiosensitivity values and (a) *COL1A1* (b) *COL5A2*, (c) *FLG*, (d) *FN1*, (e) *TNFAIP6* and (f) an extracellular matrix (ECM) signature calculated as the median expression of all aforementioned genes. Only *FN1* expression values significantly correlated (p=0.0256) with AUC radiosensitivity values.


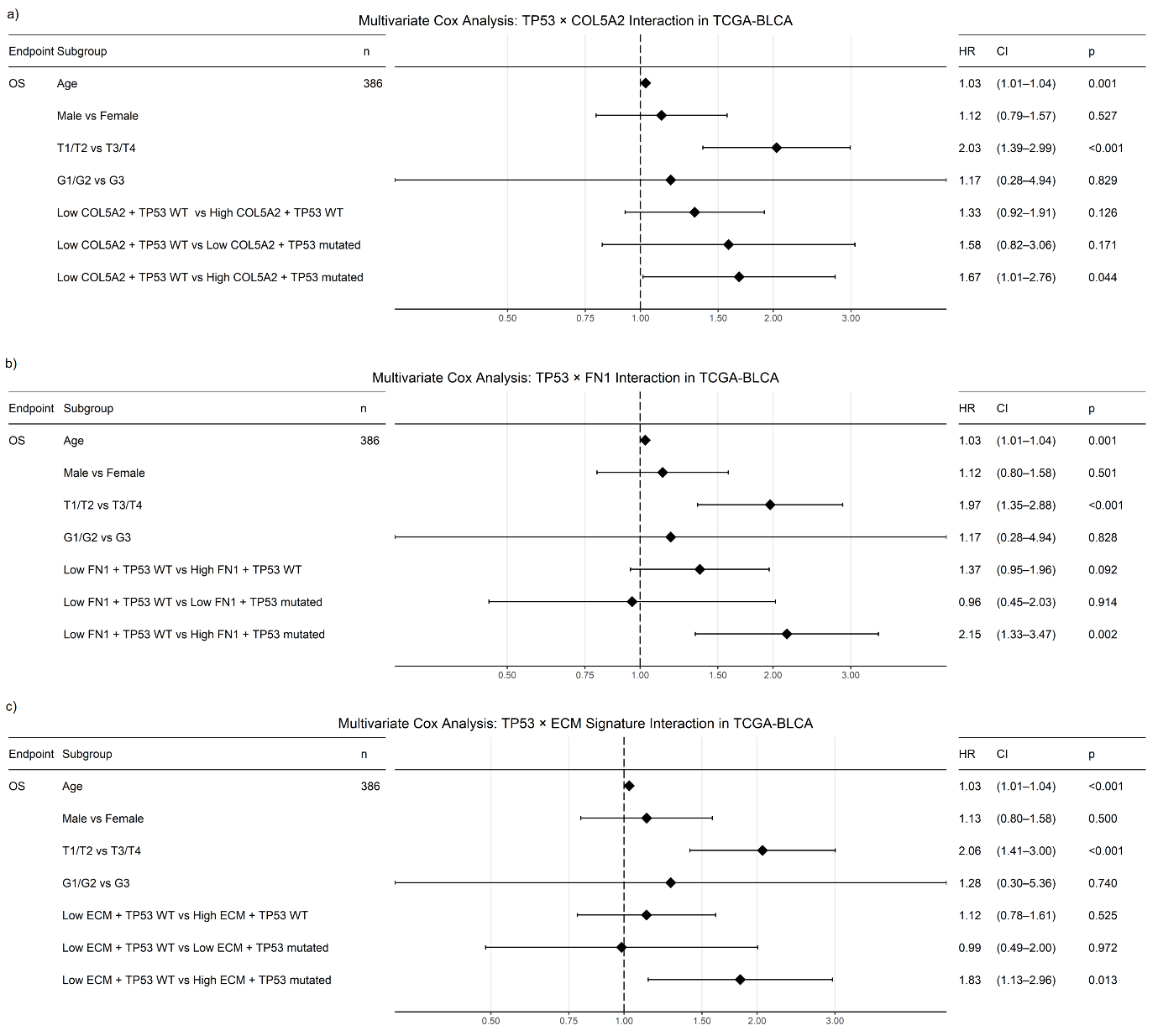


**Figure S5. TP53 mutations significantly interact with high COL5A2, FN1 expression or high ECM signature (COL1A1, COL5A2, FN1, FLG, TNFAIP6) scores, leading to poorer muscle-invasive bladder cancer (MIBC) patients prognoses.** MIBC patients with TP53 mutation and high COL5A2 (HR=1.67, p=0.044; a), high FN1 (HR=2.15, p=0.002; b), or high ECM scores (HR=1.83, p=0.013; c) have increased mortality risk independently of age, sex and stage, suggesting a relationship between TP53 status and extracellular matrix proteins expression. Analyses were conducted in a cystectomy cohort (TCGA-BLCA). Advanced age and stage were consistently adverse prognostic factors. Patients were stratified into “High” (≥50%) and “Low” (<50%) expression groups per gene. ECM scores were calculated as the median expression of signature genes, with the same dichotomisation.
